# An individual participant data analysis of prospective cohort studies on the association between subclinical thyroid dysfunction and depressive symptoms

**DOI:** 10.1038/s41598-020-75776-1

**Published:** 2020-11-05

**Authors:** Lea Wildisen, Cinzia Del Giovane, Elisavet Moutzouri, Shanthi Beglinger, Lamprini Syrogiannouli, Tinh-Hai Collet, Anne R. Cappola, Bjørn O. Åsvold, Stephan J. L. Bakker, Bu B. Yeap, Osvaldo P. Almeida, Graziano Ceresini, Robin P. F. Dullaart, Luigi Ferrucci, Hans Grabe, J. Wouter Jukema, Matthias Nauck, Stella Trompet, Henry Völzke, Rudi Westendorp, Jacobijn Gussekloo, Stefan Klöppel, Drahomir Aujesky, Douglas Bauer, Robin Peeters, Martin Feller, Nicolas Rodondi

**Affiliations:** 1 Institute of Primary Health Care (BIHAM), University of Bern, Mittelstrasse 43, 3012 Bern, Switzerland; 2 Department of General Internal Medicine, Inselspital, Bern University Hospital, University of Bern, Freiburgstrasse 15, 3010 Bern, Switzerland; 3 Service of Endocrinology, Diabetes and Metabolism, Department of Medicine, Lausanne University Hospital and University of Lausanne, Rue du Bugnon 46, 1011 Lausanne, Switzerland; 4 Division of Endocrinology, Diabetes, and Metabolism, Department of Medicine, University of Pennsylvania School of Medicine, 3400 Civic Center Boulevard, Philadelphia, PA 19104 USA; 5 K.G. Jebsen Center for Genetic Epidemiology, Department of Public Health and Nursing, NTNU, Norwegian University of Science and Technology, Postboks 8905 MTFS, 7491 Trondheim, Norway; 6 Department of Endocrinology, St. Olavs Hospital, Trondheim University Hospital, Postbox 3250 Torgarden, 7006 Trondheim, Norway; 7 Department of Internal Medicine, University Medical Center Groningen, University of Groningen, Antonius Deusinglaan 1, 9713 AV Groningen, The Netherlands; 8 Medical School, University of Western Australia Perth, The University of Western Australia (M582), 35 Stirling Highway, Crawley, WA 6009 Australia; 9 Department of Medicine and Surgery, Unit of Internal Medicine and Onco-Endocrinology, University Hospital of Parma, Via Gramsci, 14 - 43126 Parma, Italy; 10 Longitudinal Studies Section, Translational Gerontology Branch, National Institute on Aging, 251 Bayview Boulevard, Suite 100, Baltimore, MD 21224 USA; 11 Institute for Community Medicine, Clinical-Epidemiological Research, University Medicine Greifswald, Walter Rathenau Str. 48, 17475 Greifswald, Germany; 12 Department of Cardiology, Leiden University Medical Center, Postbus 9600, 2300 RC Leiden, The Netherlands; 13 Institute of Clinical Chemistry and Laboratory Medicine, University Medicine Greifswald, Ferdinand-Sauerbruch-Straße, 17475 Greifswald, Germany; 14 DZHK (German Centre for Cardiovascular Research), Partner Site Greifswald, University Medicine, Ferdinand-Sauerbruch-Straße, 17475 Greifswald, Germany; 15 Section Gerontology and Geriatrics, Department of Internal Medicine, Leiden University Medical Center, Albinusdreef 2, 2333 ZA Leiden, The Netherlands; 16 Department of Psychiatry and Psychotherapy, University Medicine Greifswald, Ellernholzstrasse 1-2, 17489 Greifswald, Germany; 17 Department of Public Health and Center for Healthy Aging, University of Copenhagen, Gothersgade 160, 1123 København K, Mærsk Tower, Copenhagen, Denmark; 18 Department of Public Health and Primary Care, Leiden University Medical Center, LUMC Education Building, Hippocratespad 21, 2333 ZD Leiden, the Netherlands; 19 University Hospital of Old Age Psychiatry, University of Bern, Murtenstrasse 21, 3008 Bern, Switzerland; 20 Departments of Medicine and Epidemiology and Biostatistics, University of California, San Francisco, 550 16th St., Box 0560, San Francisco, CA 94158 USA; 21 Department of Medicine, Erasmus Medical Center, Postbus 2040, 3000 CA Rotterdam, The Netherlands; 22 Graduate School for Health Sciences, University of Bern, Mittelstrasse 43, 3012 Bern, Switzerland

**Keywords:** Public health, Hormonal therapies, Quality of life, Psychology, Endocrinology, Medical research

## Abstract

In subclinical hypothyroidism, the presence of depressive symptoms is often a reason for starting levothyroxine treatment. However, data are conflicting on the association between subclinical thyroid dysfunction and depressive symptoms. We aimed to examine the association between subclinical thyroid dysfunction and depressive symptoms in all prospective cohorts with relevant data available. We performed a systematic review of the literature from Medline, Embase, Cumulative Index to Nursing and Allied Health Literature, and the Cochrane Library from inception to 10th May 2019. We included prospective cohorts with data on thyroid status at baseline and depressive symptoms during follow-up. The primary outcome was depressive symptoms measured at first available follow-up, expressed on the Beck’s Depression Inventory (BDI) scale (range 0–63, higher values indicate more depressive symptoms, minimal clinically important difference: 5 points). We performed a two-stage individual participant data (IPD) analysis comparing participants with subclinical hypo- or hyperthyroidism versus euthyroidism, adjusting for depressive symptoms at baseline, age, sex, education, and income (PROSPERO CRD42018091627). Six cohorts met the inclusion criteria, with IPD on 23,038 participants. Their mean age was 60 years, 65% were female, 21,025 were euthyroid, 1342 had subclinical hypothyroidism and 671 subclinical hyperthyroidism. At first available follow-up [mean 8.2 (± 4.3) years], BDI scores did not differ between participants with subclinical hypothyroidism (mean difference = 0.29, 95% confidence interval =  − 0.17 to 0.76, I^2^ = 15.6) or subclinical hyperthyroidism (− 0.10, 95% confidence interval =  − 0.67 to 0.48, I^2^ = 3.2) compared to euthyroidism. This systematic review and IPD analysis of six prospective cohort studies found no clinically relevant association between subclinical thyroid dysfunction at baseline and depressive symptoms during follow-up. The results were robust in all sensitivity and subgroup analyses. Our results are in contrast with the traditional notion that subclinical thyroid dysfunction, and subclinical hypothyroidism in particular, is associated with depressive symptoms. Consequently, our results do not support the practice of prescribing levothyroxine in patients with subclinical hypothyroidism to reduce the risk of developing depressive symptoms.

## Introduction

Subclinical thyroid dysfunction is common in the adult population and its prevalence increases with age, affecting up to 10–15% of older adults^1^. Patients are diagnosed with subclinical thyroid dysfunction when their serum thyroid-stimulating hormone (TSH) levels are below or above the reference range, but when their serum free thyroxine (fT4) levels are still within the reference range. Only a few symptoms are usually linked to subclinical thyroid dysfunction. Several guidelines discuss the association between depressive symptoms and thyroid dysfunction, and the potential benefit of levothyroxine for patients with the two diagnoses^2–5^. However, as the evidence is low, guidelines do not recommend to routinely treat patients with subclinical hypothyroidism and depressive symptoms with levothyroxine. Nevertheless, a study among GPs reported that the presence of depressive symptoms or low mood influence their decision whether or not to start treatment for subclinical hypothyroidism^6^.

The association between subclinical thyroid dysfunction and depressive symptoms is unclear because studies to date have yielded conflicting results. Several studies showed that participants with subclinical hypothyroidism or subclinical hyperthyroidism had more severe depressive symptoms, but other studies reported no differences^7–15^. The largest prospective study published showed no association between subclinical hypothyroidism and incidence of depression after 2 years of follow-up^16^, whereas depressive symptoms were associated with subclinical hyperthyroidism (but not subclinical hypothyroidism) in another prospective study^11^.

These conflicting results may be explained by differences in outcome definition and in the statistical methods used to analyse data. Individual participant data (IPD) help researchers to standardise the analyses and definitions used across studies and make it possible to identify the effects for different subgroups in large study populations^17^. For example, IPD allow the use of uniform cut-off levels of TSH to define thyroid status for each study, the same model for the analysis in each study, and stratification by age and sex.

We thus aimed to assess the association between subclinical hypothyroidism or subclinical hyperthyroidism and future development of depressive symptoms by conducting an analysis of IPD from prospective cohort studies.

## Methods

We registered this systematic review and IPD analysis in the international Prospective Register of Systematic Reviews PROSPERO (CRD42018091627) and published the study protocol^18^. This study adheres to the Preferred Reporting Items for Systematic reviews and Meta-Analyses (PRISMA) statement for IPD systematic reviews^19^.

### Search strategy and selection criteria

We performed a systematic literature search in Ovid Medline, Ovid Embase, Cumulative Index to Nursing and Allied Health Literature (CINAHL), and in the Cochrane Library, from inception to 10th May 2019. We included publications from prospective studies that measured at least baseline TSH in adults and assessed depressive symptoms during follow-up on a validated continuous depression scale or diagnosis of depression (e.g. through ICD-10 or DSM-V codes). The following search items were used: thyroid diseases, hyperthyroidism, hypothyroidism, thyroid hormones, triiodothyronine, thyroxine, thyrotropin, subclinical, sub-clinical, mild, subnormal, pre-clinical, preclinical, depression. We did not include studies of only depressed patients, pregnant women, or women wanting to get pregnant. We included studies in any language and any publication year. We worked with two experienced librarians to develop the search strategy in Ovid Medline and then translated it to match subject headings and keywords for the other databases. See Appendix 1 for details of the Medline search strategy. Details of the systematic literature search, inclusion and exclusion criteria, and IPD analysis have been described in detail elsewhere^18^. We identified additional unpublished data by contacting the Thyroid Studies Collaboration, a consortium of cohort studies that investigate the association between subclinical thyroid dysfunction and clinical outcomes^20^.

### Data extraction and quality assessment

We contacted investigators from all prospective cohort studies that met the inclusion criteria to collaborate in our IPD analysis by sharing their data. We requested IPD on thyroid status at baseline (TSH, fT4), and free triiodothyronine (fT3)), socioeconomic status (education, income), demographics (sex, age), medication use (levothyroxine, anti-thyroid, anti-depressant, thyroid-altering medication, including lithium, and amiodarone), and on depressive symptoms as measured on a validated continuous scale at baseline and at any follow-up. Each study was approved by its local ethics committee and all participants gave informed consent for the original studies. We used the Newcastle–Ottawa Scale (NOS) to assess the quality of the studies included^21^. The NOS contains eight items that focus on selection, comparability and outcome. The scale is scored from zero to nine stars; the highest score indicates the best methodological quality. We classed the studies as good, fair, and poor quality based on their star rating. We also assessed the certainty in the evidence with the GRADE tool (www.gradeworkinggroup.org). The certainty of evidence based on observational studies is «low», and may be decreased to «very low» for several reasons including study limitations (i.e. study quality), inconsistency, indirectness, imprecision, or increased for other considerations like particular design features of extremely rigorous well-conducted observational studies^22^. For assessing the study limitations, the final NOS score of the studies included in the main analysis was used. E.g. if all the included studies have a good NOS quality score the study limitations in the GRADE can be judged as “not serious” (Appendix 4).

### Thyroid function testing

Consistent with our previous IPD analyses, we used uniform TSH cut-off levels and study-specific fT4 cut-off values to define the thyroid status^20,23,24^. We defined euthyroidism as TSH levels between 0.45 and 4.50 mIU/L. Subclinical hyperthyroidism was defined as TSH levels < 0.45 mIU/L with normal fT4 values, and subclinical hypothyroidism as TSH levels > 4.50 and < 20 mIU/L and fT4 values within the reference range. For fT4, we used study specific cut-offs because these measurements show greater inter method variation than do sensitive 3rd generation TSH assays^20^. We excluded participants with fT4 values out of the reference range. When fT4 values were missing, we considered participants with TSH levels below 0.45 mIU/L to have subclinical rather than overt hyperthyroidism and participants with TSH levels above 0.45 mIU/L and below 20 mIU/L to have subclinical rather than overt hypothyroidism, because most adults with a TSH level in this range rather have subclinical than overt thyroid dysfunction^25,26^. We performed a sensitivity analysis excluding participants with missing fT4 to verify that the results remained robust, meaning that the effect size was not clinically different from the main result. We additionally performed a sensitivity analysis examining the difference in depressive symptoms between the subclinical hyperthyroidism and euthyroid participants, excluding participants with missing fT3 levels or values outside the reference range. We completed two sensitivity analyses excluding participants with thyroid medication (levothyroxine or anti-thyroid medication), and with thyroid altering medication (anti-thyroid drugs, levothyroxine, amiodarone, lithium) at baseline or follow-up.

### Depressive symptoms

Since studies used different continuous scales to measure depressive symptoms, we converted scores from different scales to the Beck Depression Inventory (BDI) scale, a commonly used depressive symptoms scale^27,28^. The BDI scale ranges from 0 to 63, with higher values indicating greater frequency and severity of depressive symptoms; the minimal clinically important difference is 5 points^28^. We calculated the conversion factor by dividing the range of the BDI scale by the range of the original scale. For example, to convert the Center for Epidemiological Studies Depression (CESD) scale to the BDI, we used a conversion factor of 1.05 (63 (BDI range) ÷ 60 (CESD range))^28^. To transfer measurements from the CESD scale to the BDI scale, we then multiplied each individual’s CESD value by the conversion factor. The primary outcome was depressive symptoms measured at first available follow-up, expressed on the BDI scale. As previously defined in the study protocol we converted measurements to the BDI scale instead of a standardised scale to facilitate the interpretation^18,28^. In a sensitivity analysis, we used the original scale to calculate the mean difference in each study and then we pooled the standardised mean differences (SMDs) across the studies. We coded SMDs so that positive values would indicate more severe depressive symptoms in participants with subclinical thyroid dysfunction than in euthyroid controls: < 0.40 was a small effect; 0.40–0.70 was a moderate effect, and > 0.70 was a large effect, respectively^29^. Since depressive symptoms could be influenced by medications, we conducted a sensitivity analysis excluding participants with antidepressant medication at baseline or follow-up. A secondary outcome was depressive symptoms at baseline, expressed on the BDI scale. An additional secondary outcome was depressive symptoms at the last available follow-up and at the third year of follow-up, expressed on the BDI scale. We chose a follow-up at year three because most of the cohorts had this follow-up time in common. Studies without a 3-year follow up were excluded from this analysis. Another secondary outcome was incidence of depressive symptoms at the first available follow-up. For the outcome of incidence of depression, we analysed data on diagnosis of depression or established cut-off points for presence of depression from the continuous depressive symptoms scales (cut-off points were defined as; (Geriatric Depression Scale 15-item (GDS-15): > 5 ^30^, CESD-20: > 21 ^31^, CESD-10: ≥ 8^32^, Hospital Anxiety and Depression Scale (HADS): > 12^33^). In the analysis of incidence of depression, we excluded participants with diagnosed depression or with depressive symptoms score above the cut-off at baseline. In the primary outcome analyses, we excluded participants with missing data on depressive symptoms at baseline or follow-up. In a sensitivity analysis, we did not exclude those participants, but used multiple imputation for missing data on depressive symptoms. We additionally conducted a sensitivity analysis excluding participants with dementia (Mini-Mental State Examination (MMSE) score < 24, or diagnosis of dementia), since the relationship between dementia and depression is complex^34^.

### Analysis

We performed a two-stage IPD analysis. In the first stage, we estimated the effect size for each study separately. For the primary outcome, we calculated the mean difference in BDI score between participants with subclinical hypothyroidism or hyperthyroidism and euthyroid controls using a multivariable linear regression model adjusted for age, sex, depressive symptoms at baseline, education, and income. We only included studies with data on depressive symptoms at baseline because adjusting for depressive symptoms at baseline adjusts for imbalance and accounts for the correlation between baseline and follow-up, which makes the effect estimates more precise^35^. In a sensitivity analysis, we additionally included studies without baseline data on depressive symptoms. When comparing incidences of depression, we calculated odds ratio for incidence of depression at the first available follow-up between subclinical hypothyroidism or hyperthyroidism and euthyroid controls using a multivariable logistic regression model adjusted for age, sex, depressive symptoms at baseline, income, and education. For the cross-sectional analysis at baseline, we calculated mean difference in BDI score between participants with subclinical hypo- or hyperthyroidism and euthyroid control, adjusted for age, sex, education, and income. In the second stage of the IPD analysis, we pooled derived mean differences or odds ratios from all the different studies from the first stage using a random effects model.

To identify sub-populations at risk and possible sources of heterogeneity, we conducted predefined subgroup and sensitivity analyses on the primary outcome. We performed predefined subgroup analyses by age (younger and older than 75 years old), by sex, by TSH levels (4.51–6.99 mIU/L, 7.00–9.99 mIU/L, 10.0–20.0 mIU/L)^20^, and by levothyroxine use at baseline.

We performed a sensitivity analysis excluding the HUNT study as this was the biggest study included in the analysis and therefore had the biggest weight for the overall result.

Heterogeneity was estimated with I^2^ and the Q test. Statistical significance testing was 2-sided and *P* < 0.05 was considered statistically significant. All analyses were conducted with Stata, release 15 (StataCorp).

### Ethics approval and consent to participate

*Statement on ethical approval from ethics committee* This is a manuscript that analysed existing cohort data. Each study in the individual participant data set received local ethical approval. Our analysis did not include identifiable data.

*Statement on guidelines followed* The manuscript adheres to the preferred reporting items for systematic reviews and meta-analyses (PRISMA) statement for IPD systematic reviews and to the PRISMA statement for systematic review protocols.

*Statement on written informed consent from the participants* This manuscript analysed existing cohort data, each study in the individual participant data set received informed consent from the participants. Our analysis did not include identifiable data.

## Results

Of the 1047 studies we identified through the literature search, four studies met our inclusion criteria (Appendix 2)^11,13,16,36^. From within the Thyroid Studies Collaboration we identified seven additional studies where data on subclinical thyroid dysfunction and depressive symptoms had not been published. We invited the investigators of eligible studies to collaborate in this IPD analysis; only one investigator, from a study identified by the literature review, declined to participate^16^. This study only presented a dichotomised result in the publication, so we could not combine this aggregate data with our main IPD analysis. We received IPD from ten studies with 33,769 participants that met our inclusion criteria. For the primary outcome analysis, we included only studies with a continuous outcome, and in which depressive symptoms had been measured at baseline. Six studies met these criteria, including 23,038 participants. Mean age (± SD) was 60 years (± 13), 65% were female, and median TSH was 1.63 mIU/L (Table 1). 21,025 (91%) participants were euthyroid, 1342 (6%) participants had subclinical hypothyroidism, and, 671 (3%) had subclinical hyperthyroidism. In sensitivity analyses, we additionally included the three studies that did not measure depressive symptoms at baseline^37–39^. For the secondary outcome of incidence of depression, we additionally included the Health in Men Study, which did not use a continuous scale to measure depressive symptoms but assessed incidence of depression via data linkage^36^. Depressive symptoms scores at baseline were balanced between groups in each cohort and on average the correlation between depressive symptoms at baseline and follow-up was 0.6 across studies (Appendix 6).

**Table 1:** Study characteristics and baseline data.

Study, Place	No. of Participants	Age, mean (range), y	Women, No. (%)	Original depression scale, (range)	Dementia, No. (%)	Thyroid medication, No. (%)	Depression medication, no. (%)	Median TSH, mIU/l	Normal range FT4, pmol/l	First/last available follow-up (mean), y
*Studies included in the main analyses*										
Leiden 85 + Study, The Netherlands	281	85 (85–85)	185 (66)	GDS-15 (0–15)	37 (13)	11 (4)	8 (3)	1.57	13–23	1.0/3.7
PROSPER Study, The Netherlands	405	75 (70–83)	190 (47)	GDS-15 (0–15)	0 (0)	10 (2)	12 (3)	2.04	12–18	3.0/3.0
InChianti Study, Italy	952	66 (21–98)	515 (54)	CESD-20 (0–60)	N.A	24 (2)	29 (3)	1.34	10–27	3.3/3.6
Health ABC Study, United States	2250	75 (69–81)	1139 (51)	CESD-20 (0–60)	481 (21)	212 (9)	7 (< 1)	2.15	10–23	2.9/7.8
CHS, United States	3419	75 (64–98)	2013 (59)	CESD-10 (0–30)	451 (13)	280 (8)	57 (2)	2.14	9–22	1.1/8.2
HUNT, Norway	15,731	53 (19–86)	10,963 (70)	HADS (0–21)	N.A	684 (4)	N.A	1.50	8–20	11.2/11.2
Overall	23,038	60 (19–98)	15,005 (65)	N.A	969 (4)	1221 (5)	113 (< 1)	1.63	N.A	8.2/9.9
*Studies only included in sensitivity analyses or secondary outcomes*										
MrOs*, United States	1356	73 (65–92)	0 (0)	GDS-15 (0–15)	29 (2)	93 (7)	71 (5)	2.04	9–24	3.9/**
PREVEND*, The Netherlands	2124	48 (28–75)	1086 (51)	N.A.^‡^	N.A	36 (2)	N.A	1.37	12–22	4.2/**
SHIP*, Germany	2139	46 (20–80)	1133 (53)	BDI (0–63)	46 (12)	105 (5)	31 (1)	0.68^§^	8–19	9.5/**
HIMS^†^, Australia	4032	77 (71–89)	0 (0)	N.A	212 (5)	126 (3)	261 (7)	2.00	10–23	N.A^¶^

*Leiden 85* + *Study*^13^ Leiden 85 plus Study; *PROSPER*^11^ prospective study of Pravastatin in the elderly at risk; *Health ABC Study*^46^ The health, aging and body composition study; *CHS*^32^ cardiovascular health study; *InChianti Study*^47^ Invecchiare in Chianti Study; *HUNT*^48^ Nord-Trøndelag Health Study; *PREVEND*^49^ prevention of renal and vascular end-stage disease; *MrOS*^50^ osteoporotic fractures in men study; *SHIP*^51^ study of health in pomerania, *HIMS*^36^ health in men study; *y* year; *GDS-15* geriatric depression scale 15-item; *CESD-10/-20* center for epidemiologic studies depression 10/20-item scale; *HADS* hospital anxiety and depression scale; *BDI* beck depression inventory scale; *TSH* thyroid stimulating hormone; *FT4* free thyroxine; *N.A.,* not available; *No.* number.

*Not included in the main analysis because no data on depressive symptoms at baseline available.

^†^Not included in the main analysis because no continuous scale used to measure depressive symptoms during follow up. Incidence of depression available (via Data Linkage).

^‡^No validated depression scale used.

^§^SHIP includes participants from Pomerania, where an iodine supplementation program began in the mid-1990s. This shifted the distribution of TSH values towards the left in its first years, which lowered TSH values in the population of the SHIP Study during baseline examinations in 1997–2001.

^¶^follow-up via data-linkage from baseline 2001–2004, censor date 2013.

**In the sensitivity analyses and subgroup analyses we only assessed the effect estimate at the first available follow-up.

### Quality assessment

Based on the NOS, the quality of all studies that we included in the primary outcome analysis was good (Appendix 3). Certainty in the evidence assessed with the GRADE tool for the primary outcome was low (Appendix 4). Because of the low number of studies, we did not assess publication bias^40^.

### Subclinical hypothyroidism

**Figure 1:**
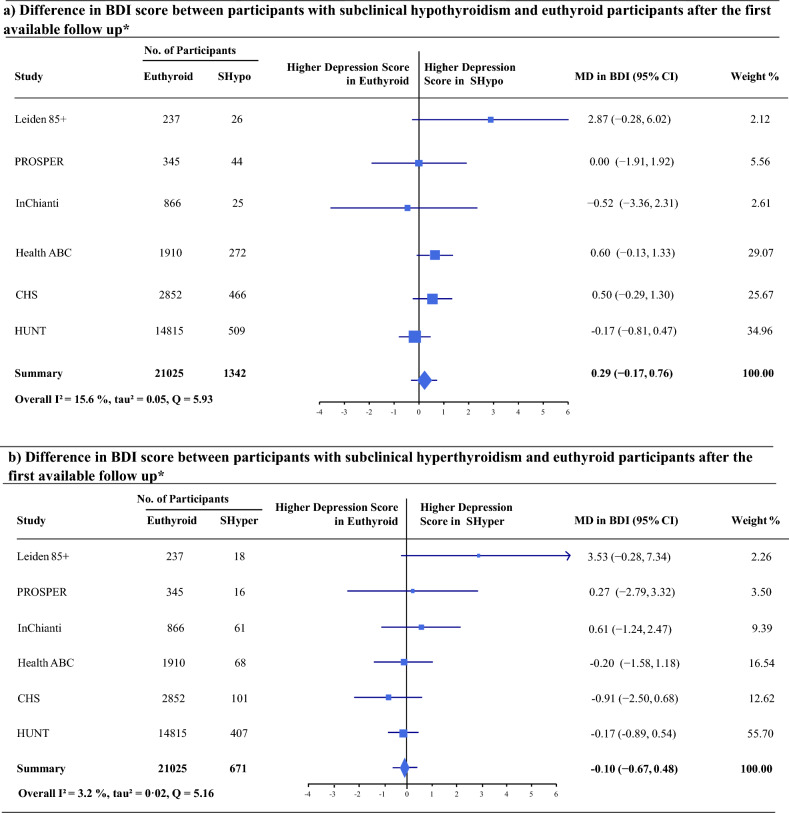
(**a**) Difference in BDI score between participants with subclinical hypothyroidism and euthyroid participants after the first available follow up*. (**b**) Difference in BDI score between participants with subclinical hyperthyroidism and euthyroid participants after the first available follow up*. * Analysis adjusted for depressive symptoms at baseline, sex, age, and education (The CHS, Health ABC Study, and the InChianti Study were additionally adjusted for income). Overall mean BDI score at first follow-up was 10.67 with a standard deviation of 8.99. Abbreviations: *SHypo* Subclinical Hypothyroidism; *SHyper* Subclinical Hyperthyroidism; *Leiden 85+* (9) Leiden 85 plus Study; *PROSPER (7)* Prospective Study of Pravastatin in the Elderly at risk; *Health ABC Study (40)* The Health, Ageing and Body Composition Study; *CHS (26)* Cardiovascular Health Study; *InChianti Study (41)* Invecchiare in Chianti Study; *HUNT (42)* Nord-Trøndelag Health Study; *BDI* Beck Depression Inventory Score (range 0–63, minimal clinically important difference 5 points); *MD* Mean Difference; *CI* Confidence Interval, *No.* Number.

At first available follow-up (mean 8.2 (± 4.3) years), there was no difference in the primary outcome of BDI score between subclinical hypothyroidism and euthyroid controls (pooled mean difference (MD) 0.29, 95% CI − 0.17 to 0.76) with a low heterogeneity (I^2^ = 15.6%) (Fig. 1).

**Figure 2:**
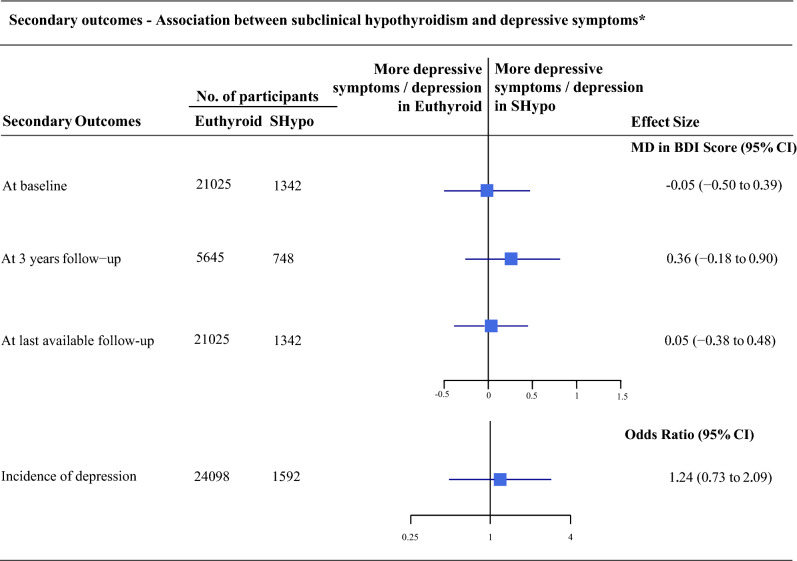
Secondary outcomes—association between subclinical hypothyroidism and depressive symptoms*. * Analysis adjusted for depressive symptoms at baseline, sex, age, and education (The CHS, Health ABC Study, and the InChianti Study were additionally adjusted for income). † Analysis includes the same studies as for the primary outcome analysis: Leiden 85+ (9), PROSPER (7), Health ABC Study (40), CHS (26), InChianti Study (41), HUNT (42). ‡ Analysis includes the same studies as in the primary outcome analysis except of HUNT (42). § Analysis includes the same studies as in the primary outcome analysis plus the HIMS (30) which only had data on incidence of depression and no continuous measurement. Abbreviations: *SHypo* Subclinical Hypothyroidism; *MD* Mean Difference; *BDI Score* Beck Depression Inventory Score (range 0–63, minimal clinically important difference 5 points); *CI* Confidence Interval;* No.* Number.

Secondary outcomes are shown in Fig. 2. At baseline there was neither relevant difference in depressive symptoms between euthyroid participants (mean BDI = 10.28) and participants with subclinical hypothyroidism (mean BDI = 9.63), nor in multivariable analysis adjusted for age, sex, education, and income (MD in BDI scores − 0.05 95% CI =  − 0.50–0.39 I^2^ = 0.0%). In the five cohorts (N = 6393) with data on depressive symptoms at 3 years follow-up, BDI scores did not differ between participants with subclinical hypothyroidism and euthyroid controls (MD 0.36, 95% CI − 0.18–0.90, I^2^ = 0.0%). At last available follow-up (9.9 ± 3.3 years) there was no association in BDI score between subclinical hypothyroidism and euthyroid control (MD 0.05, 95% CI − 0.38–0.48, I^2^ = 0.0%). The odds ratio for incident depression comparing subclinical hypothyroid and euthyroid participants was 1.24 (95% CI 0.73–2.09, I^2^ = 56.4%).

**Figure 3:**
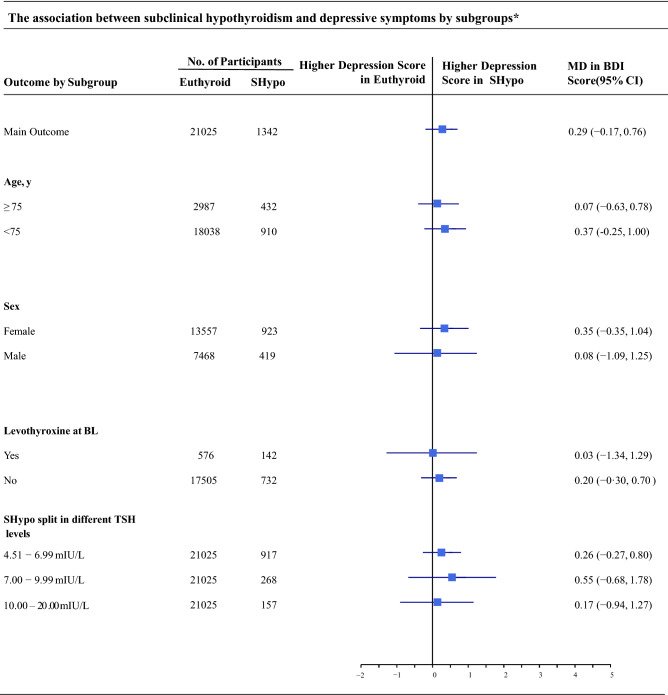
The association between subclinical hypothyroidism and depressive symptoms by subgroups*. * Analysis adjusted for depressive symptoms at baseline, sex, age, and education (The CHS, Health ABC Study, and the InChianti Study were additionally adjusted for income). Abbreviations: *SHypo* Subclinical Hypothyroidism; *TSH* Thyroid-Stimulating Hormone; *MD* Mean Difference; *BDI* *Score* Beck Depression Inventory Score (range 0–63, minimal clinically important difference 5 points); *CI* Confidence Interval; *No.* Number.

Results of sensitivity analyses in which we excluded participants taking thyroid medication (N = 21,391), thyroid altering medication (N = 21,384) or antidepressant medication (N = 23,175), participants with dementia (N = 22,224), or participants without fT4 measurements at baseline (N = 5618), or in which we excluded the study with the biggest weight (HUNT) (N = 7043), were similar comparable to those from our primary analysis (Table 2). When we used multiple imputation, depressive symptoms between subclinical hypothyroidism and euthyroidism did also not differ in a sensitivity analysis that included participants with t4 data on outcome and depressive symptoms at baseline (N = 45,398). Results of a sensitivity analysis in which we included studies without baseline information on depressive symptoms (N = 27,361) were similar to those of the main analysis. In a sensitivity analysis that used the original scale from each study, we found SMD between groups of 0.04 (95% CI =  − 0.02–0.09). Figure 3 shows the results of several subgroup analyses. After stratifying the population that was included in the primary outcome analysis by age (participants older and younger than 75), by sex, by levothyroxine treatment at baseline, and by different TSH levels, we found no significant differences in depressive symptoms scores.

**Table 2:** Sensitivity analyses on subclinical hypothyroidism and depressive symptoms at first available follow-up*

Sensitivity analysis	No. of participants		No. of included studies^†^	MD in BDI score (95% CI), I^2^
	Euthyroid	SHypo		
Main analysis	21,025	1342	6	0.29 (− 0.17–0.76) ^§^, 15.6%
1) Excluding participants with thyroid medication	20,268	1123	6	0.30 (− 0.15–0.75), 4.7%
2) Excluding participants with thyroid altering medication	20,261	1123	6	0.30 (− 0.15–0.76), 4.8%
3) Excluding participants with antidepressant medication	20,897	1327	6	0.32 (− 0.19–0.83), 26.1%
4) Excluding participants with dementia	20,203	1222	6	0.24 (− 0.17–0.65), 0.0%
5) Excluding participants without FT4 measurement	4550	1068	5	0.17 (− 0.49–0.83), 11.8%
6) Using multiple imputation to impute missing outcome data	42,759	2639	6	0.35 (− 0.10–0.81), 10.7%
7) Including studies without depressive symptoms at baseline	25,851	1510	9	0.25 (− 0.13–0.63), 0.0%
8) Not adjusted for income	21,509	1413	6	0.22 (− 0.22, 0.65), 11.1%
9) Excluding HUNT	6210	833	5	0.54 (0.04, 1.05), 0.0%
			SMD in depressive symptoms (95% CI), I^2^	
10) Using original scale for depressive symptoms^‡^	21,025	1342	6	0.04 (− 0.02–0.09), 27.2%

*SHypo* subclinical hypothyroidism; *TSH* thyroid-stimulating hormone; *FT4* free thyroxine; *MD* mean difference; *SMD* standardised mean difference; *BDI Score* Beck DEPRESSION INVENTORY SCORE (range 0–63, minimal clinically important difference 5 points); *CI* confidence Interval; No number.

*Analyses adjusted for depressive symptoms at baseline (In sensitivity analysis 7: except studies without measurement), sex, age, income, and education (The CHS, Health ABC Study, and the InChianti Study were additionally adjusted for income).

^†^The main analysis includes the same studies as for the primary outcome analysis: Leiden 85 + ^13^, PROSPER^11^, Health ABC Study^46^, CHS^32^, InChianti Study^47^, HUNT^48^.

**Sensitivity analyses 1–4, 6, 8, 10**: the same studies as in the main analysis were included, only participants with a certain measurement missing were excluded.

**Sensitivity analysis 5:** the same studies as in the main analysis without the Health ABC Study ^32^ because in this study FT4 was not measured in the euthyroid group.

**Sensitivity analysis 7:** the same studies as in the main analysis plus 3 studies that did not have data for depressive symptoms at baseline were included (PREVEND (Prevention of Renal and Vascular end-stage Disease)^49^, MrOS (Osteoporotic Fractures in Men)^50^, SHIP (Study of Health in Pomerania)^51^).

**Sensitivity analysis 9:** the same studies as in the main analysis without the HUNT^48^ study as the HUNT study has the biggest weight in the summarized result of the main outcome (34.96%).

^‡^Mean differences using the original scale for depressive symptoms within each study were pooled.

^§^Overall mean BDI score at first follow-up of all 23,038 participants was 10.67 with a standard deviation of 8.97.

### Subclinical Hyperthyroidism

There was no difference in the primary outcome of depressive symptoms between subclinical hyperthyroidism and euthyroid controls (MD 0.10, 95% CI − 0.67–0.48, I^2^ = 3.2%) at first available follow-up (Fig. 1), as well as at year three follow-up, and at last available follow-up (Appendix 5a). At baseline, there was no difference in depressive symptoms expressed on the BDI scale between euthyroid participants (Mean BDI = 10.26) and participants with subclinical hyperthyroidism (Mean BDI = 10.28) (Appendix 5). Odds for incidence of depression were not higher for participants with subclinical hyperthyroidism than for euthyroid controls (Appendix 5a). Our results remained robust in several sensitivity and subgroup analyses (Appendix 1b,c).

## Discussion

In this IPD analysis of 23,038 participants from six prospective cohort studies, we found no clinically relevant differences in depressive symptoms during follow-up between subclinical hypothyroidism or hyperthyroidism and euthyroid controls. Depressive symptoms of participants with subclinical hypothyroidism or hyperthyroidism were not different from symptoms of euthyroid control participants at baseline or at any follow-up. Participants with subclinical hypothyroidism were not at increased risk for incidence of depression. Our results were robust across all sensitivity analyses.

To our knowledge, no pooled IPD analysis has previously assessed the association of subclinical thyroid dysfunction and depressive symptoms. The results are in contrast with two previous meta-analyses of cross-sectional studies^7, 8^ which found a positive association between subclinical hypothyroidism and depression. Reasons for difference in results could be that we included published and unpublished studies, that we did not include studies only on depressed patients, that we did not include case–control studies and cross-sectional studies (only cross-sectional analysis of prospective studies in our analysis, as they are considered of higher validity), and that we analyzed individual participant data, which leads to far more reliable results^17,35^. With individual participant data, we could use standardized definition of subclinical hypo- and hyperthyroidism for all studies, which was not possible in these study-level meta-analyses. Our results are in line with the results found by the Kangbuk Samsung Health Study showing that participants with subclinical hypothyroidism had no higher incidence of depression than euthyroid controls^16^.

Our study has some limitations. First, younger people were underrepresented because three of six studies included participants only over 64. However, our sensitivity analysis that excluded participants over 75 also yielded no association, but we were able to include too few participants to assess risk among middle-aged adults. Second, we were limited by measurement of depressive symptoms using different scales across studies, so that we were unable to combine the effect estimates from different studies using their original scales. To standardise the scale between studies, we converted scores from the various scales to the BDI scale, a common scale whose scores are easy to interpret^28^. As there was no validated conversion factor, we examined whether our results remained robust when we converted the original scores to a standardised scale, which yielded similar findings. Third, we did not have access to information about treatment prior to baseline; patients with subclinical thyroid dysfunction and depressive symptoms may have been more frequently diagnosed with subclinical thyroid dysfunction and been treated to restore euthyroidism prior to baseline, in which case the subclinical thyroid dysfunction group at baseline would overrepresent the number of people who did not develop depressive symptoms. Fourth, the diagnosis of subclinical hypothyroidism was based on one assessment of TSH, and did not depend on a second verified measurement, which is a limitation of most published large cohorts that have examined the risk of subclinical thyroid dysfunction^20,41^. Based on a single elevated or decreased TSH level, participants might revert to normal thyroid function over follow-up, which could have biased the results towards the null. However, previous IPD analyses including cohorts with just one TSH assessment documented an association between subclinical thyroid dysfunction and both coronary heart disease or fractures^20,24^. Inferring a causal relationship from observational data is challenging and, for this reason, we completed a series of sensitivity analysis to minimise the potential effects of residual confounding and bias.

Our study was strengthened by its IPD analysis, considered the most appropriate method in evidence synthesis since it offers many advantages over aggregate data analysis^42^. For example, our results do not suffer from the ecological bias of study-level meta-analyses. We could also standardise definitions of predictors and outcomes, use uniform adjustments for potential confounders to reduce heterogeneity across studies, and include unpublished data to increase the robustness of our results and our power to detect associations. Because our IPD analysis was large, we could assess the effects of age, sex, thyroid medication, antidepressant medication, and TSH levels in subgroup analyses.

Current guidelines for the management of people with subclinical hypothyroidism tend to recommend thyroid hormone substitution for adults with TSH levels > 10 mIU/L and for people with lower TSH values who are young or symptomatic, although some recent guidelines have more narrow indications^43,44^. As we found no association between subclinical hypothyroidism and depressive symptoms, one might infer that thyroid hormones would be of limited benefit for the treatment of depressive symptoms in affected people with subclinical hypothyroidism. This is in line with a previous meta-analysis of four small randomised clinical trials (total N = 278) that found no positive association between thyroid hormone therapy and depressive symptoms^45^. Overall, our results do not support increased risk of depressive symptoms in adults with subclinical thyroid dysfunction.

## Supplementary information

Supplementary Information

## Data Availability

The datasets analysed during this study are available from the corresponding author on reasonable request.

## Abbreviations

BDI: Beck depression inventory
CESD: Center for epidemiological studies depression
CI: Confidence interval
CINAHL: Cumulative index to nursing and allied health literature
DSM-5: Diagnostic and statistical manual of mental disorders, fifth edition
fT3: Free triiodothyronine
fT4: Free thyroxine
GDS: Geriatric depression scale
HADS: Hospital anxiety and depression scale
ICD: International statistical classification of diseases and related health problems
IPD: Individual participant data/individual patient data
MD: Mean difference
MMSE: Mini-mental state examination
N: Number
NOS: Newcastle–Ottawa scale
PRISMA: Preferred reporting items for systematic reviews and meta-analyses
PROSPERO: International prospective register of systematic reviews
SD: Standard deviation
SHyper: Subclinical hyperthyroidism
SHypo: Subclinical hypothyroidism
SMD: Standardized mean difference
TSH: Thyroid-stimulating hormone
y: Year
