# Supplementary material for: An individual participant data analysis of prospective cohort studies on the association between subclinical thyroid dysfunction and depressive symptoms
Source: Sci Rep. 2020 Nov 5;10:19111. doi: 10.1038/s41598-020-75776-1 (PMC7644764; doi:10.1038/s41598-020-75776-1)
Supplement: Supplementary file 1 — Supplementary Information [file 41598_2020_75776_MOESM1_ESM.docx]

# **An Individual Participant Data Analysis of Prospective Cohort Studies on the association between Subclinical Thyroid Dysfunction and Depressive Symptoms**

Lea Wildisen MSc*^1^, Cinzia Del Giovane PhD^1^, Elisavet Moutzouri MD^1,2^, Shanthi Beglinger MBChB^1,2^, Lamprini Syrogiannouli PhD^1^, Tinh-Hai Collet, MD^3^; Anne R. Cappola MD^4^, Bjørn O. Åsvold MD^5,6^, Stephan J.L. Bakker MD^7^, Bu B. Yeap PhD^8^, Osvaldo P. Almeida MD^8^, Graziano Ceresini MD^9^, Robin P.F. Dullaart MD^7^, Luigi Ferrucci MD^10^, Hans Grabe MD^11^, J. Wouter Jukema MD^12^, Matthias Nauck MD^13,14^, Stella Trompet PhD^15^, Henry Völzke MD^16^ Rudi Westendorp MD^17^, Jacobijn Gussekloo MD^15,18^, Stefan Klöppel MD^19^, Drahomir Aujesky MD^2^, Douglas Bauer MD^20^, Robin Peeters MD^21^, Martin Feller MD^1,2^ Nicolas Rodondi MD^1,2^.

Author Affiliations

^1^Institute of Primary Health Care (BIHAM), University of Bern, Bern, Switzerland (Mittelstrasse 43, 3012 Bern, Switzerland);

^2^Department of General Internal Medicine, Inselspital, Bern University Hospital, University of Bern, Bern, Switzerland (Freiburgstrasse 15, 3010 Bern, Switzerland);

^3^Service of Endocrinology, Diabetes and Metabolism, Department of Medicine, Lausanne University Hospital and University of Lausanne, Switzerland (Rue du Bugnon 46, 1011 Lausanne, Switzerland);

^4^Division of Endocrinology, Diabetes, and Metabolism, Department of Medicine, University of Pennsylvania School of Medicine, Philadelphia, Pennsylvania, United States (3400 Civic Center Boulevard, Philadelphia, PA 19104, United States);

^5^K.G. Jebsen Center for Genetic Epidemiology, Department of Public Health and Nursing, NTNU, Norwegian University of Science and Technology, Trondheim, Norway (Postboks 8905 MTFS, NO-7491 Trondheim, Norway);

^6^Department of Endocrinology, St. Olavs Hospital, Trondheim University Hospital, Trondheim, Norway (Postbox 3250 Torgarden, NO-7006 Trondheim, Norway);

^7^Department of Internal Medicine, University Medical Center Groningen, University of Groningen, Groningen, the Netherlands (Antonius Deusinglaan 1, 9713 AV Groningen, The Netherlands);

^8^Medical School, University of Western Australia Perth, Western Australia, Australia (The University of Western Australia (M582), 35 Stirling Highway, Crawley WA 6009, Australia);

^9^Department of Medicine and Surgery, Unit of Internal Medicine and Onco-Endocrinology, University Hospital of Parma, Parma, Italy (Via Gramsci, 14 - 43126 Parma, Italy);

^10^Longitudinal Studies Section, Translational Gerontology Branch, National Institute on Aging, Baltimore, Maryland, United States (251 Bayview Boulevard, Suite 100, Baltimore, MD 21224, United States);

^11^Institute for Community Medicine, Clinical-Epidemiological Research, University Medicine Greifswald, Greifswald, Germany (Walter Rathenau Str. 48, 17475 Greifswald, Germany);

^12^Department of Cardiology, Leiden University Medical Center, Leiden, the Netherlands (Postbus 9600, 2300 RC Leiden, The Netherlands);

^13^Institute of Clinical Chemistry and Laboratory Medicine, University Medicine Greifswald, Greifswald, Germany (Ferdinand-Sauerbruch-Straße, 17475 Greifswald, Germany);

^14^DZHK (German Centre for Cardiovascular Research), Partner Site Greifswald, University Medicine, Greifswald, Germany (Ferdinand-Sauerbruch-Straße 17475 Greifswald, Germany)

^15^Section Gerontology and Geriatrics, Department of Internal Medicine, Leiden University Medical Center, Leiden, the Netherlands (Albinusdreef 2, 2333 ZA Leiden, the Netherlands);

^16^Department of Psychiatry and Psychotherapy, University Medicine Greifswald, Greifswald, Germany (Ellernholzstrasse 1-2, 17489 Greifswald, Germany);

^17^Department of Public Health and Center for Healthy Aging, University of Copenhagen, Copenhagen, Denmark (Gothersgade 160, 1123 København K, Mærsk Tower, Denmark);

^18^Department of Public Health and Primary Care, Leiden University Medical Center, Leiden, the Netherlands ([LUMC Education Building](https://www.universiteitleiden.nl/en/locations/lumc-building-3), Hippocratespad 21 2333 ZD Leiden, the Netherlands);

^19^University Hospital of Old Age Psychiatry, University of Bern, Bern, Switzerland (Murtenstrasse 21, 3008 Bern, Switzerland);

^20^Departments of Medicine and Epidemiology & Biostatistics, University of California, San Francisco, California, United States (Box 0560, 550 16th St., San Francisco, CA 94158, United States);

^21^Department of Medicine, Erasmus Medical Center, Rotterdam, the Netherlands (Postbus 2040, 3000 CA Rotterdam, the Netherlands);

**Corresponding author**: Lea Wildisen, Institute of Primary Health Care (BIHAM), University of Bern, Mittelstrasse 43, 3012 Bern, Switzerland; Phone +41 31 631 58 71, email: lea.wildisen@biham.unibe.ch

## Appendix

### Appendix 1: Search strategy for Medline (May 10, 2019)

Medline (via Ovid)

1 thyroid diseases/ or hyperthyroidism/ or hypothyroidism/ or thyroid hormones/ or triiodothyronine/ or thyroxine/ or exp Thyrotropin/

2 (subclinical or sub-clinical or mild or subnormal or pre-clinical or preclinical).ti,ab,kw.

3 1 and 2

4 ((subclinical or sub-clinical or mild or subnormal or pre-clinical or preclinical) adj6 (hypothyroid* or hyperthyroid* or thyroid dysfunction* or tri?odothyronin* or thyroxin*or TSH or T4 or T3 or thyroid failure* or thyroid disease*)).ti,ab,kw.

5 ((thyroid function adj6 normal range*) or (TSH adj6 normal range*) or (TSH adj6 range)).ti,ab,kw.

6 4 or 5

7 3 or 6

8 Depression/

9 (depression* or depressive* or depressed* or mood disorder* or (low adj1 mood)).ti,ab,kw.

10 8 or 9

11 7 and 10

12 exp animals/ not humans/

13 11 not 12

### Appendix 2: Study Selection, Flow diagram of the systematic review


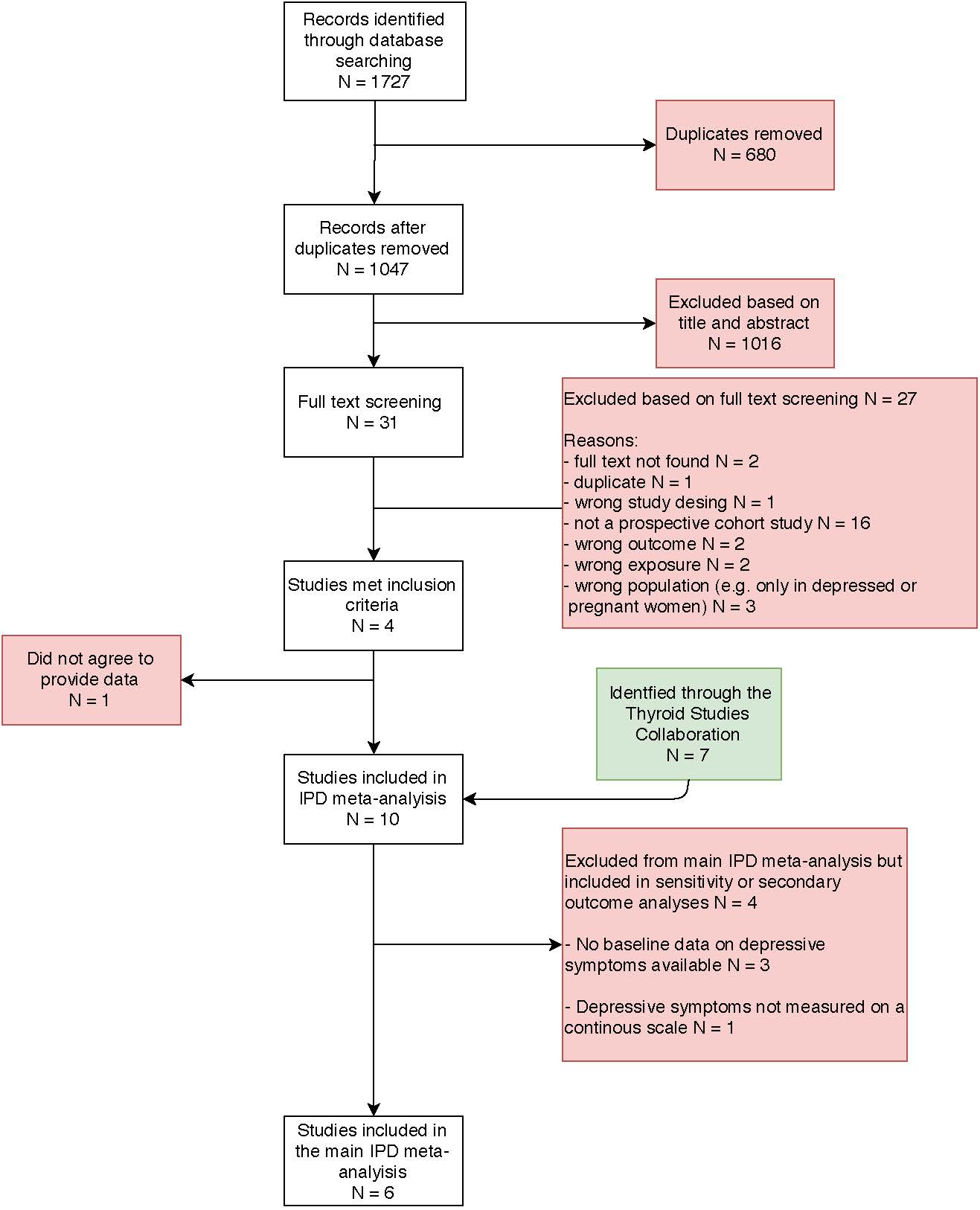


### Appendix 3: Quality assessment using the Newcastle-Ottawa quality assessment scale for cohort studies

The first category refers to the representativeness of the study. The criterion was met if conducted in the general population, including both sexes, and adults in each age. The second category refers to selection of the non-exposed. Criterion was met if studies had euthyroid controls. The third category refers to ascertainment of exposure. The criterion was met if studies used an appropriate TSH measurement. The fourth category refers to demonstration that outcome of interest was not present at start of the study. Criterion was met if depressive symptoms were measured at baseline. The fifth category refers to comparability on the basis of the design or analysis. Criterion was met when the co-variables on age and sex were available. The sixth category refers to assessment of the outcome, criterion was met if depressive symptoms were measured with a validated depression score at follow up. The seventh category refers to the length of follow-up. Criterion was met if the follow-up was at least one year. The category eight refers on the adequacy of follow-up, criterion was met if the loss of follow up was ≤ 15%. We considered the final NOS quality score (Appendix 3) of studies for judging the study limitations (risk of bias) in the GRADE assessment. E.g., all the six studies included in the main analysis had a good NOS quality score. Therefore, we classified the study limitations in the GRADE tool as “not serious” (Appendix 4).

| Study | Selection | | | | Comparability | Outcome | | |  |
| --- | --- | --- | --- | --- | --- | --- | --- | --- | --- |
|  | **Representativeness of the exposed cohort** | **Selection of the non exposed cohort** | **Ascertainment of exposure** | **Demonstration that outcome of interest was not present at start of the study** | **Comparability of cohorts on the basis of the design or analysis** | **Assessment of outcome** | **Was follow-up long enough for outcomes to occur** | **Adequacy of follow up (Loss of follow-up)*** | **Quality Score** |
| Leiden 85+ Study | Everyone at age 85 from Leiden | Yes ★ | Third generation TSH assay ★ | Yes ★ | age, sex, education ★ | GDS-15 ★ | Yes ★  5 years | 15% Loss of follow-up ★ | 7 Stars  Good quality |
| PROSPER Study | Participants with pre-existing  cardiovascular or cerebrovascular disease or at high risk of developing  such a disease | Yes ★ | Third generation TSH assay ★ | Yes ★ | age, sex, education ★ | GDS-15 ★ | Yes ★  3 years | 15 % loss to follow-up ★ | 7 Stars  Good quality |
| Health ABC Study | Medicare-eligible adults residing in the areas surrounding Pittsburgh, Pennsylvania and Memphis, TN. Aged 70-79 | Yes ★ | Third generation TSH assay ★ | Yes ★ | age, sex, income, education ★★ | CES-D ★ | Yes ★  10 years | 6% loss to follow-up ★ | 8 Stars  Good quality |
| Cardiovascular Health Study | Population based (>65 years old) | Yes ★ | Third generation TSH assay ★ | Yes ★ | age, sex, income, education ★★ | CES-D ★ | Yes ★  23 years | 5% loss to follow-up ★ | 8 Star  Good quality |
| Invecchiare in  Chianti Study (InChianti) | Population based (> 65 years of age) | Yes ★ | Third generation TSH assay ★ | Yes ★ | age, sex, income, education ★★ | CES-D ★ | Yes ★  9 years | 11% loss to follow-up ★ | 7 Stars  Good quality |
| Nord-Trøndelag Health Study (HUNT) | all women >40 years, a random 50% sample of men >50 years and random 5% samples of women and men 20-40 years from the Nord-Trøndelag county★ | Yes ★ | Third generation TSH assay ★ | Yes ★ | age, sex, education ★ | HADS ★ | Yes ★  10 years | 49% loss to follow-up | 7 Stars  Good quality |
| PREVEND | all inhabitants of city of Groningen, the Netherlands, aged 28-75 ★ | Yes ★ | Third generation TSH assay ★ | No (no baseline data for outcome) | Age, sex ★ | No validated questionnaire | Yes ★  6 years | 8% loss to follow-up ★ | 6 Stars  Good quality |
| Osteoporotic  Fractures in  Men (MrOS) Study | Only men, population based | Yes ★ | Third generation TSH assay ★ | No (no baseline data for outcome) | Age, sex, education ★ | GDS-15 ★ | Yes ★  14 years | 13 % loss to follow-up ★ | 6 Stars  Fair quality |
| Study of  Health in  Pomerania (SHIP) | Population based (Legend: all from ship00 were invited); TSH levels shifted to the left, because of iodine supplementation | Yes ★ | Third generation TSH assay ★ | No (no baseline data for outcome) | Age, sex, education, income ★★ | BDI ★ | Yes ★  9 years | 3% loss to follow-up ★ | 7 Stars  Fair quality |
| Health In Men Study (HIMS) | population-based; Only men aged over 65 years | Yes ★ | Third generation TSH assay ★ | Yes ★ | Age, sex, education ★ | Diagnosis of depression ★ | Yes ★ | Data linkage (estimated loss to follow-up <2%) ★ | 7 Stars  Good quality |

*at the first available follow-up (primary outcome)

Thresholds for converting the Newcastle-Ottawa scales to AHRQ standards (good, fair, and poor): Good quality: 3 or 4 stars in selection domain AND 1 or 2 stars in comparability domain AND 2 or 3 stars in outcome/exposure domain Fair quality: 2 stars in selection domain AND 1 or 2 stars in comparability domain AND 2 or 3 stars in outcome/exposure domain Poor quality: 0 or 1 star in selection domain OR 0 stars in comparability domain OR 0 or 1 stars in outcome/exposure domain.

Abbreviations: **PROSPER**, Prospective Study of Pravastatin in the Elderly at risk; **Health ABC Study**, The Health, Ageing and Body Composition Study; **PREVEND**, Prevention of Renal and Vascular end-stage Disease; **GDS-15**, Geriatric Depression Scale 15 item; **CES-D**, Center for Epidemiologic Studies Depression; **HADS**, Hospital Anxiety and Depression Scale; **BDI**, Beck Depression Inventory Scale,

### Appendix 4: Grading of Recommendation Assessment, Development and Evaluation (GRADE)

**Question**: Participants with subclinical Hypo- or hyperthyroidism compared to euthyroid controls for depressive symptoms during follow-up

| **Certainty assessment** | | | | | | | **№ of patients** | | **Effect** | | **Certainty** | |
| --- | --- | --- | --- | --- | --- | --- | --- | --- | --- | --- | --- | --- |
| **№ of studies** | **Study design** | **Risk of bias/ Study Limitations** | **Inconsistency** | **Indirectness** | **Imprecision** | **Other considerations** | **Exposed Group** | **Control Group** | | **Absolute (95% CI)** |  |  |
| **Depressive Symptoms (SHypo) (assessed with: Beck Depression Inventory Scale; Scale from: 0 to 63)** | | | | | | | | | | | |  |
| 6 | observational studies | not serious ^a^ | not serious | not serious | not serious | none | 1463 | 21894 | | MD **0.29 higher** (0.17 lower to 0.76 higher) | ⨁⨁◯◯ LOW | |
| **Depressive Symptoms (SHyper) (assessed with: Beck Depression Inventory Scale; Scale from: 0 to 63)** | | | | | | | | | | | |  |
| 6 | observational studies | not serious | not serious | not serious | not serious | none | 708 | 21894 | | MD **0.1 lower** (0.67 lower to 0.48 higher) | ⨁⨁◯◯ LOW | |

**CI:** Confidence interval; **MD:** Mean difference; **OR:** Odds ratio; Explanations: a. blinding; №; Number; **Certainty:** **High,** Further research is very unlikely to change our confidence int the estimate of effect, **Moderate:** Further research is likely to have an important impact on our confidence in the estimate of effect and may change the estimate. **Low:** Further research is very likely to have an important impact on our confidence in the estimate of effect and is likely to change the estimate, **Very low:** Any estimate of effect is very uncertain

### Appendix 5: Subclinical hyperthyroidism and depressive symptoms

**5a) Secondary outcomes – Association between subclinical hyperthyroidism and depressive symptoms ***

|  | No. of participants with SHyper | No. of euthyroid participants | Effect Size (95% CI), I^2^ |
| --- | --- | --- | --- |
| At baseline^†^ | 671 | 21025 | MD = 0·12 (-0·66, 0.90), 17·6% |
| At 3 years follow-up^‡^ | 243 | 5645 | MD = 0·46 (-0·40, 1·33), 0·0% |
| At last available follow-up^†^ | 671 | 21025 | MD = 0·02 (-0·30, 0·43), 0·0% |
| Incidence of depression^§^ | 712 | 24098 | OR = 1·06 (0·69, 1·64), 0·0% |

*Analysis adjusted for depressive symptoms at baseline, sex, age, and education (The CHS, Health ABC Study, and the InChianti Study were additionally adjusted for income).

^†^  Analysis includes the same studies as for the primary outcome analysis: Leiden 85+ (9), PROSPER (7), Health ABC Study (40), CHS (Cardiovascular Health Study) (26), InChianti Study (Invecchiare in Chianti Study) (41), HUNT (Nord-Trøndelag Health Study) (42).

^‡^ Analysis includes the same studies as in the primary outcome analysis except of HUNT (Nord-Trøndelag Health Study) (42).

^§^ Analysis includes the same studies as in the primary outcome analysis plus the HIMS (Health in Men Study) (30) which only had data on incidence of depression and no continuous measurement.

Abbreviations: **SHyper**, Subclinical Hyperthyroidism; **MD**, Mean Difference in Beck Depression Inventory Score (range 0-63, minimal clinically important difference 5 points); **OR**, Odds Ratio for having depression; **CI**, Confidence Interval; **No.**, Number

**5b) Sensitivity analysis for the association between subclinical hyperthyroidism and depressive symptoms at the first available follow-up***

|  | No. of participants with SHyper | No. of euthyroid participants | No. of included studies | Effect Size (95% CI), I^2^ |
| --- | --- | --- | --- | --- |
| Main Outcome^†^ | 671 | 21025 | 6 | MD = -0·10 (-0·67, 0·48), 3·2% |
| 1) Exclude participants with thyroid medication | 402 | 20268 | 6 | MD = 0·19 (-0·50, 0·89), 0·0% |
| 2) Exclude particpants with thyroid-altering medication | 402 | 20261 | 6 | MD = 0·20 (-0·50, 0·89), 0·0% |
| 3) Exclude participants with antidepressant medication | 664 | 20897 | 6 | MD = -0·05 (-0·77, 0·68), 23·9% |
| 4) Exclude participants with dementia | 644 | 20203 | 6 | MD = -0·09 (-65, 0·46), 0·0% |
| 5) Exclude participants without FT4 measurements | 375 | 4550 | 5 | MD = 0·34 (-0·72, 1·40), 23·9% |
| 6) Exclude participants without or abnormal FT3 measurement | 211 | 1044 | 2 | MD = 1.12 (-0.64, 2.88), 0.0% |
| 7) Using multiple imputatio to impute missing outcome data | 1395 | 42759 | 6 | MD = -0·11 (-0·73, 0·52), 0·0% |
| 8) Not adjusted for income | 694 | 21509 | 6 | MD = -0.01 (-0·63, 0·61), 11·0% |
| 9) Including studies without depressive symptoms at baseline | 1319 | 25851 | 9 | MD = 0·07 (-0·36, 0·49), 0·0% |
| 10) Excluding HUNT study | 264 | 6210 | 5 | MD = 0.07 (-0.93 to 1.07), 21·3% |
| 11) Using original scale^‡^ | 671 | 21025 | 6 | SMD = -0·01 (-0·05, 0·04), 4·6% |

*Analyses adjusted for depressive symptoms at baseline (In sensitivity analysis 7: except studies without measurement), sex, age, income, and education (The CHS, Health ABC Study, and the InChianti Study were additionally adjusted for income).

^†^ The main analysis includes the same studies as for the primary outcome analysis: Leiden 85+ (9), PROSPER (7), Health ABC Study (40), CHS (Cardiovascular Health Study) (26), InChianti Study (Invecchiare in Chianti Study) (41), HUNT (Nord-Trøndelag Health Study) (42).

**Sensitivity analyses 1-4, 7-8, 11**: the same studies as in the main analysis were included, only participants with a certain measurement missing were excluded.

**Sensitivity analysis 5:** The same studies as in the main analysis without the Health ABC Study (26), because this study did not measure FT4 in the euthyroid group.

**Sensitivity analysis 6:** includes the InChianti Study (41) und Leiden85+ Study (9).

**Sensitivity analysis 9:** the same studies as in the main analysis plus 3 studies that did not have data for depressive symptoms at baseline were included (PREVEND (Prevention of Renal and Vascular end-stage Disease) (43), MrOS (Osteoporotic Fractures in Men) (44), SHIP (Study of Health in Pomerania) (45)).

**Sensitivity analysis 10**: same studies as in the main analysis without HUNT (Nord-Trøndelag Health Study) (42), as the HUNT study has the biggest weight in the summarized result of the main outcome (55.70 %).

^‡^ Mean differences using the original scale for depressive symptoms within each study were pooled.

Abbreviations: **SHyper**, Subclinical Hyperthyroidism; **TSH**, Thyroid-Stimulating Hormone; **FT4**, Free Thyroxine, **MD**, Mean Difference in Beck Depression Inventory Score (range 0-63, minimal clinically important difference 5 points); **SMD**, Standardised Mean Difference; **CI**, Confidence Interval.

**5c) Subgroup analysis - Association between subclinical hyperthyroidism and depressive symptoms by subgroups ***

|  | No. of participants with SHyper | No. of euthyroid participants | Mean Difference in BDI (95% CI), I^2^ |
| --- | --- | --- | --- |
| **Main Outcome** | 671 | 21025 | -0·10 (-0·67, 0·48), 3·2% |
| **Age** |  |  |  |
| ≥75 | 129 | 2987 | 0·02 (-1·33, 1·37), 5·5% |
| < 75 | 542 | 18038 | -0·16 (-0·78, 0·45), 0·0% |
| **Sex** |  |  |  |
| Female | 525 | 13557 | 0·01 (-0·62, 0·63), 0·0% |
| Male | 146 | 7468 | -0·46 (-1·90, 0·98), 29·1% |
| **Thyroxine at baseline** |  |  |  |
| Yes | 201 | 576 | -0·42 (-2·25, 1·42), 32·4% |
| No | 365 | 17505 | 0·11 (-0·63, 0·84), 0·0% |
| **SHyper split in different TSH Levels** |  |  |  |
| > 0.10 mIU/L | 201 | 21025 | -0·27 (-1·49, 0·96), 18·8% |
| 0.10 – 0.45 mIU/L | 470 | 21025 | 0·09 (-0·88, 1·05), 32.8% |

* Analysis adjusted for depressive symptoms at baseline, sex, age, and education (The CHS, Health ABC Study, and the InChianti Study were additionally adjusted for income).

Abbreviations: **SHyper**, Subclinical Hyperthyroidism; **BDI**, Beck Depression Inventory Score (range 0-63, minimal clinically important difference 5 points); **CI**, Confidence Interval; **No.**, Number.

| Study | Number of Participants | | | Transferred Scale (Range)^*^  Original Scale (Range) | Mean Baseline Depressive Symptoms Score (SD) | | | Mean FUP Depressive Symptoms Score (SD) | | |
| --- | --- | --- | --- | --- | --- | --- | --- | --- | --- | --- |
|  | SHypo | Euthyroid | SHyper |  | SHypo | Euthyroid | SHyper | SHypo | Euthyroid | SHyper |
| Leiden 85+ | 26 | 237 | 18 | BDI (0-63) | 8·2 (9·3) | 9·7 (10·0) | 9·6 (10·7) | 11·6 (10·4) | 9·8 (10·8) | 13·3 (14·8) |
|  |  |  |  | GDS-15 (0-15) | 2·0 (2·2) | 2·3 (2·4) | 2·3 (2·5) | 2·8 (2·5) | 2·3 (2·6) | 3·2 (3·5) |
| PROSPER | 44 | 345 | 16 | BDI (0-63) | 9·2 (6·3) | 10·0 (7·8) | 7·0 (5·4) | 9·0 (7·4) | 9·9 (8·3) | 17·7 (6·6) |
|  |  |  |  | GDS-15 (0-15) | 4·4 (3·0) | 4·8 (3·7) | 3·3 (2·5) | 4·3 (3·5) | 4·7 (4·0) | 3·7 (3·1) |
| InChianti | 25 | 866 | 61 | BDI (0-63) | 15·4 (12·1) | 12·2 (8·6) | 11·2 (6·6) | 17·1 (9·8) | 15·0 (8·8) | 15·8 (9·1) |
|  |  |  |  | CESD-20 (0-60) | 14·7 (11·5) | 11·6 (8·2) | 10·7 (6·3) | 16·4 (9·3) | 14·.2 (8·4) | 15·0 (8·6) |
| Health ABC | 275 | 1910 | 68 | BDI (0-63) | 4·8 (5·7) | 4·8 (5·5) | 5·4 (6·0) | 7·3 (7·8) | 6·7 (6·5) | 7·0 (6·8) |
|  |  |  |  | CESD-20 (0-60) | 4·6 (5·4) | 4·6 (5·2) | 5·1 (5·7) | 6·9 (7·5) | 6·4 (6·2) | 6·7 (6·5) |
| CHS | 466 | 2852 | 101 | BDI (0-63) | 11·0 (9·7) | 11·2 (10·2) | 11·7 (9·6) | 11·4 (10·8) | 11·0 (10·3) | 10·6 (8·1) |
|  |  |  |  | CESD-10 (0-30) | 5·2 (4·6) | 5·3 (4·9) | 5·6 (4·6) | 5·4 (5·2) | 5·3 (4·9) | 5·0 (3·8) |
| HUNT | 509 | 14815 | 407 | BDI (0-63) | 10·9 (8·6) | 10·7 (8·9) | 11·4 (9·4) | 11·0 (8·6) | 10·9 (8·7) | 11·2 (9·0) |
|  |  |  |  | HADS (0-21) | 3·6 (2·9) | 3.6 (3·0) | 3·8 (3·1) | 3·6 (2·9) | 3.6 (2·9) | 3·7 (3·0) |

### Appendix 6: Depressive symptoms scores in different thyroid status groups at baseline and follow-up

Abbreviations: **SHypo**, Subclinical Hypothyroidism; **SHyper**, Subclinical Hyperthyroidism; **FUP**, First available follow-up; **Leiden 85+** (9), Leiden 85 plus Study; **PROSPER** (7), Prospective Study of Pravastatin in the Elderly at risk; **Health ABC Study** (40), The Health, Ageing and Body Composition Study; **CHS** (26), Cardiovascular Health Study; **InChianti Study** (41), Invecchiare in Chianti Study; **HUNT** (42), Nord-Trøndelag Health Study; **BDI**, Beck Depression Inventory Score (range 0-63, minimal clinically important difference 5 points); **GDS-15**, Geriatric Depression Scale 15-item; **CESD-10/-20**, Center for Epidemiologic Studies Depression 10/20-item Scale; **HADS**, Hospital Anxiety and Depression Scale; **SD**, Standard Deviation. ^*^ Scores from the original depression scale were transferred to the BDI scale by multiplying the original scores by a converting factor. The converting factor was calculated by dividing the range of the BDI scale with the range of the original scale.
